# Editorial: Reviews in microbial pathogenesis

**DOI:** 10.3389/fmicb.2025.1568840

**Published:** 2025-02-28

**Authors:** Axel Cloeckaert, Daniel Pletzer, Svetlana Khaiboullina

**Affiliations:** ^1^INRAE, Université de Tours, UMR, ISP, Nouzilly, France; ^2^Department of Microbiology and Immunology, University of Otago, Dunedin, New Zealand; ^3^Department of Microbiology and Immunology, University of Nevada, Reno, NV, United States

**Keywords:** microbiology, review, infectious disease, pathogens, microbial pathogenesis, virulence

Microbiology is a fast-growing research field. The rapid expansion of this field is driven by ongoing discoveries of emerging pathogens, microbial adaptation, resistance mechanisms and technological advancements. While these trends will likely continue to drive new discoveries and novel solutions for infectious diseases, the overwhelming volume of research publications makes it difficult for scientists to keep pace with the latest findings. Additionally, access to cutting-edge research can be limited by paywalls, creating barriers for both early-career researchers and those in resource-limited settings.

The present Research Topic, focusing on reviews in microbial pathogenesis, includes 16 publications (15 peer-reviewed articles and 1 erratum), including 72 authors, to address these challenges. By providing open-access, comprehensive reviews and accessible summaries of recent findings, this Research Topic offers a valuable platform for the researchers to navigate key advancements in the field of microbiology, emphasizing important directions for future research and offer insights into emerging trends and methodologies.

The review by Mohammad et al. addressed the role of lipoproteins (Lpps) in the pathogenesis of *Staphylococcus aureus*. *S. aureus* produces Lpps that contribute to metabolism, are essential for the survival of bacteria and are important for tissue invasion. Additionally, they are capable of affecting disease pathogenesis by modulating the immune response (Mohammad et al., 2020; Nguyen and Götz, 2016) via binding to Toll-Like Receptors 2 (TLR2), leading to activation of innate immune responses (Hashimoto et al., 2006). Binding of these molecules to TLR2 causes rapid migration of innate immune cells, including monocytes/macrophages and neutrophils (Mohammad et al., 2019, 2021). Also, Lpps can stimulate interferon γ producing T cells (Saito and Quadery, 2018), although there is limited effect on B cell activation (Mohammad et al., 2019). The *in vitro* and *in vivo* effects of Lpps are believed to be mediated by interaction with TLR2-dependent neutrophil recruitment (Mohammad et al., 2019). It was demonstrated that neutrophils and macrophage recruitment is facilitated by the release of chemokines such as MIP-2, KC, MCP-1 and MPO (Mohammad et al., 2021). The role of TLR2 in the pathogenesis of Lpps-induced pathology is also supported by the TLR2-knockout mouse model (Schmaler et al., 2009).

In another review, Schwermann and Winstel summarize the functional diversity of *Staphylococcus* surface proteins, which play a crucial role in host interaction and pathogenesis. *Staphylococcus* expresses several surface proteins, including clumping factor B (ClfB), fibronetin-binding protein B (FnBPB), and iron-regulated surface determinant protein A (IsdA), which bind to human loricrin receptor on epithelial cells. This binding facilitates initial adhesion and invasion of host cells (Clarke et al., 2009; da Costa et al., 2022; Mulcahy et al., 2012), particularly on skin and nasal surfaces. Bacterial surface proteins also contribute to immune evasion and persistence (Kim et al., 2010). For example, Clumping factor A(ClfA), collagen adhesin and protein A (SpA) are key factors in the pathogenesis of septic arthritis (Josefsson et al., 2001; Palmqvist et al., 2002; Xu et al., 2004). ClfA is also involved in biofilm formation, which protects *S. aureus* from phagocytosis (Dastgheyb et al., 2015). The essential role of *S. aureus* surface proteins in infections make them attractive targets for the development of novel therapeutics. Immunization with IsdA or IsdB has been shown to reduce the virulence of *S. aureus* (Kim et al., 2010). Additionally, anti-SpA monoclonal antibodies demonstrated therapeutic potential in mouse abscess models (Cheng et al., 2011). Overall, this strongly indicates that targeting specific surface proteins could be a viable strategy for controlling *S. aureus* infections.

Acosta-Espana and Voigt shed light into the differences between entomophthoromycosis and mucormycosis. Fungi, causing entomophthoromycosis and mucormycosis, were initially placed into the class *Zygomycetes* (Voigt et al., 1999), including *Entomophthorales* and *Mucorales*. In 2007, the phylum *Zygomycetes* was replaced by *Mucoromycota and Zoopagomycota* (Spatafora et al., 2016). However, the old terms are still used in many publications, creating confusion about fungal species identification. The authors summarize information on *Basidiobolales, Entomophthorales*, and *Mucorales* to address this confusing issue and make it clear and easy for clinical researchers to use the updated fungal taxonomy. The authors state that current taxonomy identifies the classes *Mucoromycota* (order *Mucorales), Zoopagomycota* (order *Entomophthorales* [*Conidiobolus* spp.]) and *Basidiobolales* (*Basidiobolus* spp.). Instead of the term “zygomycosis”, more defined terms should be used, such as: 1. Infection with *Mucorales* should be referred to as mucormycosis; 2. Infection with *Basidiobolus* spp. as basidiobolomycosis; 3. *Conidiobolus* spp. as conidiobolomycosis. These fungal infections have differences in pathogenesis as mucormycosis is diagnosed primarily in patients with impaired cellular immunity [Center for Disease and Prevenstion (CDC), n.d.], while basidiobolomycosis and conidiobolomycosis occur in immunocompetent patients (Geramizadeh et al., 2015; Kundu and Chakraborty, 2023; Spatafora et al., 2016). Clinical presentations of these infections differ as well. Patients infected with *Mucorales* species have rapid spread with angioinvasion and necrosis [Center for Disease and Prevenstion (CDC), n.d.]. In contrast, the slow progression of clinical symptoms is characteristic of *Basidiobolus* spp. and *Conidiobolus* spp. (Raghavan et al., 2020). The diagnosis is based on epidemiologic, clinical, imaging, histopathologic, microbiologic, and molecular data, followed by the confirmatory report of a fungal culture.

A review by Mlynek and Bozue addressed the impact of phase variation and biofilm formation in *Francisella tularensis*. *F. tularensis* causes tularemia, a zoonotic disease often transmitted through contact with rabbits (Ellis et al., 2002). There are two primary subspecies: *F. tularensis subsp. tularensis* (Type A) and *F. tularensis subsp. holarctica* (Type B), both can be transmitted to humans (Larson et al., 2020). Different species within the *Francisella* genus exhibit varying capabilities to form biofilms. Subspecies of *F. tularensis* tend to form less defined structures compared to *Francisella novicida* (Mahajan et al., 2011; Margolis et al., 2010). These differences are partly due to genetic variations. For example, *F. novicida* retains a functional cyclic-di-GMP system (c-di-GMP), which is absent in *F. tularensis* (Kingry and Petersen, 2014). The *wbt* locus in *F. tularensis*, which contributes to O antigen synthesis, contains genes that are not present in *F. novicida* (Kingry and Petersen, 2014). GMP stimulates biofilm formation by upregulating genes encoding extracellular polysaccharides (Hickman et al., 2005). Additionally, the O antigen contributes to biofilm formation in *F. tularensis* isolates (Champion et al., 2019). Biofilms enhance bacterial persistence by protecting against host defenses and antibiotic treatment. Notably, biofilm formation has been shown to reduce susceptibility of *F. tularensis* to ciprofloxacin (Siebert et al., 2020), further complicating treatment efforts.

The role of *Francisella* peptidoglycan biosynthesis enzymes in morphology, pathogenesis and treatment of infection is discussed in the review by Bachert and Bozue. The bacterial cell wall is constantly remodeling in response to environmental changes and cell division. Peptidoglycan (PG) remodeling is a coordinated process involving several enzymes. PG biosynthesis begins with the formation of a lipid II precursor (Egan et al., 2020). The precursor is subsequently polymerized with penicillin-binding proteins (PBPs). Many organisms encode multiple PG enzymes with redundant function (Lee et al., 2017; van Heijenoort, 2011). Interestingly, this is not a characteristic of *F. tularensis* (Kijek et al., 2019). There are currently five carboxypeptidases and two lytic transglycosylases known in *Francisella* (Sauvage et al., 2008). They all have distinct roles in cell morphology (Spidlova et al., 2018; Zellner et al., 2021) and contribute to the immunomodulating activity of this bacterium (Nakamura et al., 2021). This suggests that PG enzymes could be used as a therapeutic target specifically against this organism.

Approaches for identifying bacterial effector kinases are summarized in the review by Louis et al. Many pathogens encode proteins with sequence homology to eukaryotic kinase domains (Anderson et al., 2015; Moss et al., 2019; Navarro et al., 2007). Some of these bacterial kinases can phosphorylate host cell proteins to manipulate signaling pathways, thereby promoting bacterial replication and survival within the host (Park et al., 2019; Tegtmeyer et al., 2017). However, understanding the role of these kinases in the pathogenesis of bacterial disease is limited, primarily due to insufficient knowledge of their target host proteins. Improved identification of host targets for bacterial kinases could pave the way for the development of novel antimicrobial therapeutics that disrupt these critical interactions.

Manipulation of host signaling pathways by *Neisseria gonorrhoeae* is discussed by Walker et al. The mucosal epithelium serves as the primary portal of entry for *N. gonorrhoeae* (Quillin and Seifert, 2018). During colonization, bacterial pili facilitate cell-to-cell contact with the epithelium, while Opa proteins further promote adherence. Gonococci pili bind to several host receptors, including α1β1 and α2β1 integrins in the male urethral epithelium (Edwards and Apicella, 2005). In contrast, gonococcal pili can also bind to the complement receptors, CD46 and CR3, as well as the I-domain containing integrin receptors (Edwards et al., 2001). The interaction between microbial Opa protein and the CEACAM family of receptors on neutrophils contributes to the clinical manifestation of gonococcal infection (Sarantis and Gray-Owen, 2012). The CEACAM-Opa interaction promotes the colonization of cervical epithelial cells by suppressing exfoliation (Yu et al., 2019). Furthermore, CEACAM-Opa inhibits Th1/Th2 lymphocyte responses while promoting a pro-inflammatory Th17 lymphocyte phenotype (Feinen et al., 2010; Liu et al., 2012). Another key gonococcal protein, PorB, increases calcium influx, which is required to reduce lysosome counts in infected cells (Hopper et al., 2000). PorB also reprograms macrophages (Mosleh et al., 1998) to create a more favorable environment for survival. Additionally, PorB can bind to soluble C4b-binding protein (C4BP) and factor H of complement (Ram et al., 1998, 2001), aiding immune evasion. Understanding the mechanisms employed by *N. gonorrhoeae* to evade immune clearance and promote intracellular replication is essential for the development of vaccines and therapeutics.

The importance of interaction between host and microbial neuraminidases (NA) in the pathogenesis of viral and bacterial co-infection of respiratory epithelium is discussed by Escuret and Terrier. Pathogens infecting epithelial cells of the respiratory tract such as influenza viruses use NA and hemagglutinin (HA) to enter the cell. Bacteria can also express NA for adherence and invasion of epithelial cells (Vimr and Lichtensteiger, 2002). Intriguingly, during viral-bacterial co-infections, viral NA can remove sialic acids that typically mask bacterial adhesion receptors, thereby facilitating bacterial colonization (Peltola and McCullers, 2004). This synergistic effect enhances the severity of respiratory infections (Wren et al., 2017). Given the pivotal role of NA in viral and bacterial interactions, they present attractive targets for developing preventive and therapeutic strategies aimed at mitigating co-infection severity.

Jin et al. discussed the advancements in the understanding of mechanisms of *Bartonella* pathogenesis. Endothelial cells are the primary target for *Bartonella* species (Deng et al., 2012). The bacterium uses α-enolase or phosphopyruvate hydratase to activate plasmin and promote extracellular matrix degradation (Díaz-Ramos et al., 2012). The *Bartonella* BadA protein can activate hypoxia-inducible factor-1 and secrete pro-angiogenic cytokines (Kempf et al., 2001, 2005). It can also provide resistance to complement killing (Deng et al., 2012). BadA and Vomp proteins also facilitate immune evasion by antigen variations (Linke et al., 2006). To evade the immune repose, *Bartonella* produces LPS, an antagonist of the TLR4 receptor (Malgorzata-Miller et al., 2016). Still, many aspects of *Bartonella*'s pathogenesis remain unknown, requiring the development of novel *in vivo* and *in vitro* methods.

Recent data on pathogens causing sepsis are summarized in the review by Gatica et al. A diverse group of pathogens that belong to the normal microflora (*Escherichia coli, Klebsiella pneumoniae, Staphylococcus aureus, Pseudomonas aeruginosa*, and *Streptococcus pyogenes*) can cause sepsis (Gouel-Cheron et al., 2022). Sepsis risk increases with age, compromised immunocompetence and comorbidities. Also, each microbe has a unique set of virulence factors facilitating adhesion, penetration and replication. Therefore, approaches for diagnosis and treatment will differ for each type of sepsis. The traditional use of antibiotics led to the development of drug-resistant strains, making therapeutic options limited. Therefore, searching for new approaches for diagnosing and treating sepsis remains a pressing medical issue.

In the review by Ayesha et al. the role of *Legionella pneumophila* outer membrane vesicles (OMVs) in interaction with the host is discussed. *L. pneumophila* secretes OMVs containing proteins, toxins, nucleic acids and antibiotic-resistance enzymes (Flesher et al., 1979). OMV cargo delivered to eukaryotic cells can inhibit innate protection against bacteria. For example, it was shown that proteins delivered by OMVs can inhibit the fusion of legionella-containing phagosomes and lysosomes (Fernandez-Moreira et al., 2006). Also, OMVs can inhibit the production of pro-inflammatory cytokines by macrophages (Jung et al., 2016). The ability of OMVs to deliver the cargo could be used to develop vaccines and deliver drugs.

The role of infection in Kawasaki vasculitis is discussed in the review by Wang et al. Environmental factors were suggested to play a role in the disease pathogenesis (Chang et al., 2020). However, the seasonal nature of outbreaks suggests an infectious etiology of Kawasaki vasculitis (Valtuille et al., 2023). Multiple DNA and RNA viruses and bacterial pathogens were suggested as causing Kawasaki vasculitis (Guo et al., 2022; Huang et al., 2020; Kafetzis et al., 2001; Xiao et al., 2020). Having many microbes linked to Kawasaki vasculitis could indicate that the disease is multifactorial, where multiple factors contribute to the disease pathogenesis.

The role of lipolytic enzymes in the pathogenesis of *Mycobacterium tuberculosis* is discussed in the review by Lin et al. There are four types of lipolytic enzymes in *M. tuberculosis* (Mtb) based on specificity to a substrate (Dedieu et al., 2013; Delorme et al., 2012) The first class contains lipases hydrolyzing water-insoluble long-chain carboxylesters like long-chain triglycerides (TAG). Esterases are in the second group, which hydrolyze small water-soluble carboxylesters. The third group includes phospholipases. The last four groups contain cutinases, which digest carboxylesters. Lipases digest lipids in the extracellular matrix, promoting Mtb tissue penetration (Nazarova et al., 2017). Also, Mtb lipases digest lipids to release energy and survive inside the cells (Kumari et al., 2020). Mtb lipases could be used as a disease biomarker (Low et al., 2009) or could be a target for novel therapeutics (West et al., 2011).

The interaction between microflora and cervical cancer progression is discussed by Amaris et al. Cervical cancer is ranked as the most common cancer in women (Arbyn et al., 2020). *Fusobacterium* spp*., Peptostreptococcus* spp.*, Campylobacter* spp.*, and Haemophilus* spp., as potential biomarkers for cervical cancer progression (He et al., 2022; Wu et al., 2021; Zhou et al., 2022). Additionally, *Alloscardovia* spp.*, Eubacterium* spp.*, and Mycoplasma* spp. were identified in HPV-positive cervical cancer (Gao et al., 2013), while *Methylobacterium* spp. were more often detected in HPV-negative carcinomas.

Animal models of *Klebsiella pneumonia* infection of the mucosa are summarized by Assoni et al. Multiple factors should be considered when selecting an animal model: site of infection, type of immune response and susceptibility of an animal. Mice and rats were the most used to study *K. pneumonia* respiratory tract infection (Ferreira et al., 2019; van der Weide et al., 2020). A rabbit model was used to study empyema caused by *K. pneumonia* (Shohet et al., 1987). More recently, cynomolgus macaques were used to study the pathogenesis and immune response to *K. pneumonia* (Liu et al., 2022). This model provides an inside look at the immune response to this microbe. The Zebrafish model was used to study neutrophil and macrophage reaction to *K. pneumonia* (Zhang et al., 2019). *K. pneumonia* can colonize different niches, which makes it challenging to select an appropriate animal model. Careful considerations should be taken before selecting a model to study *K. pneumonia* infection.

In conclusion, this Research Topic provides a collection of reviews covering pathogenic mechanisms of some important microbial pathogens of the section Infectious Agents and Disease. This Research Topic will be of interest for researchers, healthcare providers and infection control officials.

## References

[B1] Anderson D. M. Feix J. B. Frank D. W. (2015). Cross Kingdom Activators of Five Classes of Bacterial Effectors. PLoS Pathog. 11:e1004944. 10.1371/journal.ppat.1004944 26203905 PMC4512716

[B2] Arbyn M. Weiderpass E. Bruni L. de Sanjos,é S. Saraiya M. Ferlay J.. (2020). Estimates of incidence and mortality of cervical cancer in 2018: a worldwide analysis. Lancet Glob. Health 8, e191–e203. 10.1016/S2214-109X(19)30482-6 31812369 PMC7025157

[B3] Center for Disease and Prevenstion (CDC) (n.d.). “Clinical overview of mucormycosis,” in Fungal Diseases.

[B4] Champion A. E. Catanzaro K. C. F. Bandara A. B. Inzana T. J. (2019). Formation of the *Francisella tularensis* biofilm is affected by cell surface glycosylation, growth medium, and a glucan exopolysaccharide. Sci. Rep. 9:12252. 10.1038/s41598-019-48697-x 31439876 PMC6706388

[B5] Chang L.-S. Yan J.-H. Li J.-Y. Yeter D. Des Y. Huang Y.-H.. (2020). Blood mercury levels in children with kawasaki disease and disease outcome. Int. J. Env. Res. Public Health 17, 3726. 10.3390/ijerph17103726 32466179 PMC7277186

[B6] Cheng A. G. DeDent A. C. Schneewind O. Missiakas D. (2011). A play in four acts: *Staphylococcus aureus* abscess formation. Trends Microbiol. 19, 225–232. 10.1016/j.tim.2011.01.007 21353779 PMC3087859

[B7] Clarke S. R. Andre G. Walsh E. J. Dufrêne Y. F. Foster T. J. Foster S. J. (2009). Iron-regulated surface determinant protein A mediates adhesion of *Staphylococcus aureus* to human corneocyte envelope proteins. Infect. Immun. 77, 2408–2416. 10.1128/IAI.01304-08 19307218 PMC2687344

[B8] da Costa T. M. Viljoen A. Towell A. M. Dufrêne Y. F. Geoghegan J. A. (2022). Fibronectin binding protein B binds to loricrin and promotes corneocyte adhesion by *Staphylococcus aureus*. Nat. Commun. 13:2517. 10.1038/s41467-022-30271-1 35523796 PMC9076634

[B9] Dastgheyb S. Parvizi J. Shapiro I. M. Hickok N. J. Otto M. (2015). Effect of biofilms on recalcitrance of staphylococcal joint infection to antibiotic treatment. J. Infect. Dis. 211, 641–650. 10.1093/infdis/jiu514 25214518 PMC4318921

[B10] Dedieu L. Serveau-Avesque C. Kremer L. Canaan S. (2013). Mycobacterial lipolytic enzymes: a gold mine for tuberculosis research. Biochimie 95, 66–73. 10.1016/j.biochi.2012.07.008 22819994

[B11] Delorme V. Diomand,é S. V. Dedieu L. Cavalier J.-F. Carrière F. Kremer L.. (2012). MmPPOX inhibits *Mycobacterium tuberculosis* lipolytic enzymes belonging to the hormone-sensitive lipase family and alters mycobacterial growth. *PLoS ONE* 7 :e46493. 10.1371/journal.pone.0046493 23029536 PMC3460867

[B12] Deng H. Le Rhun D. Buffet J.-P. R. Cotté V. Read A. Birtles R. J.. (2012). Strategies of exploitation of mammalian reservoirs by *Bartonella* species. Vet. Res. 43:15. 10.1186/1297-9716-43-15 22369683 PMC3430587

[B13] Díaz-Ramos À. Roig-Borrellas A. García-Melero A. López-Alemany R. (2012). α -Enolase, a multifunctional protein: its Role on pathophysiological situations. J. Biomed. Biotechnol. 2012, 1–12. 10.1155/2012/156795 23118496 PMC3479624

[B14] Edwards J. L. Apicella M. A. (2005). I-domain-containing integrins serve as pilus receptors for *Neisseria gonorrhoeae* adherence to human epithelial cells. Cell. Microbiol., 7, 1197–1211. 10.1111/j.1462-5822.2005.00547.x 16008586

[B15] Edwards J. L. Brown E. J. Ault K. A. Apicella M. A. (2001). The role of complement receptor 3 (CR3) in *Neisseria gonorrhoeae* infection of human cervical epithelia. Cell. Microbiol. 3, 611–622. 10.1046/j.1462-5822.2001.00140.x 11553013

[B16] Egan A. J. F. Errington J. Vollmer W. (2020). Regulation of peptidoglycan synthesis and remodelling. Nat. Rev. Microbiol. 18, 446–460. 10.1038/s41579-020-0366-3 32424210

[B17] Ellis J. Oyston P. C. F. Green M. Titball R. W. (2002). Tularemia. Clin. Microbiol. Rev. 15, 631–646. 10.1128/CMR.15.4.631-646.2002 12364373 PMC126859

[B18] Feinen B. Jerse A. E. Gaffen S. L. Russell M. W. (2010). Critical role of Th17 responses in a murine model of *Neisseria gonorrhoeae* genital infection. Mucosal Immunol. 3, 312–321. 10.1038/mi.2009.139 20107432 PMC2857675

[B19] Fernandez-Moreira E. Helbig J. H. Swanson M. S. (2006). Membrane vesicles shed by *Legionella pneumophila* inhibit fusion of phagosomes with lysosomes. Infect. Immun. 74, 3285–3295. 10.1128/IAI.01382-05 16714556 PMC1479291

[B20] Ferreira R. L. da Silva B. C. M. Rezende G. S. Nakamura-Silva R. Pitondo-Silva A. Campanini E. B.. (2019). High prevalence of multidrug-resistant *Klebsiella pneumoniae* harboring several virulence and β-lactamase encoding genes in a brazilian intensive care unit. Front. Microbiol. 9:3198 10.3389/fmicb.2018.03198 30723463 PMC6349766

[B21] Flesher A. R. Ito S. Mansheim B. J. Kasper D. L. (1979). The cell envelope of the Legionnaires' disease bacterium. Ann. Intern. Med. 90:628. 10.7326/0003-4819-90-4-628 434648

[B22] Gao W. Weng J. Gao Y. Chen X. (2013). Comparison of the vaginal microbiota diversity of women with and without human papillomavirus infection: a cross-sectional study. BMC Infect. Dis. 13:271. 10.1186/1471-2334-13-271 23758857 PMC3684509

[B23] Geramizadeh B. Heidari M. Shekarkhar G. (2015). Gastrointestinal Basidiobolomycosis, a Rare and Under-diagnosed Fungal Infection in Immunocompetent Hosts: a Review Article. Iran. J. Med. Sci. 40, 90–97. 25821287 PMC4359942

[B24] Gouel-Cheron A. Swihart B. J. Warner S. Mathew L. Strich J. R. Mancera A.. (2022). Epidemiology of ICU-onset bloodstream infection: prevalence, pathogens, and risk factors among 150,948 ICU patients at 85 U.S. Hospitals. Crit Care Med. 50, 1725–1736. 10.1097/CCM.0000000000005662 36190259 PMC10829879

[B25] Guo M. M.-H. Yang K. D. Liu S.-F. Kuo H.-C. (2022). Number of kawasaki disease admissions is associated with number of domestic COVID-19 and severe enterovirus case numbers in Taiwan. Children 9:149. 10.3390/children9020149 35204870 PMC8870605

[B26] Hashimoto M. Tawaratsumida K. Kariya H. Aoyama K. Tamura T. Suda Y. (2006). Lipoprotein is a predominant Toll-like receptor 2 ligand in *Staphylococcus aureus* cell wall components. Int. Immunol. 18, 355–362. 10.1093/intimm/dxh374 16373361

[B27] He Z. Tian W. Wei Q. Xu J. (2022). Involvement of *Fusobacterium nucleatum* in malignancies except for colorectal cancer: a literature review. Front. Immunol. 13:968649. 10.3389/fimmu.2022.968649 36059542 PMC9428792

[B28] Hickman J. W. Tifrea D. F. Harwood C. S. (2005). A chemosensory system that regulates biofilm formation through modulation of cyclic diguanylate levels. Proc. Natl. Acad. Sci. USA. 102, 14422–14427. 10.1073/pnas.0507170102 16186483 PMC1234902

[B29] Hopper S. Vasquez B. Merz A. Clary S. Wilbur J. S. So M. (2000). Effects of the immunoglobulin A1 protease on *Neisseria gonorrhoeae* trafficking across polarized T84 epithelial monolayers. Infect. Immun. 68, 906–911. 10.1128/IAI.68.2.906-911.2000 10639461 PMC97220

[B30] Huang S.-H. Chen C.-Y. Weng K.-P. Chien K.-J. Hung Y.-M. Hsieh K.-S.. (2020). Adenovirus infection and subsequent risk of Kawasaki disease: a population-based cohort study. J. Chin. Med. Assoc. 83, 302–306. 10.1097/JCMA.0000000000000266 31990817 PMC13048015

[B31] Josefsson E. Hartford O. O'Brien L. Patti J. M. Foster T. (2001). Protection against experimental *Staphylococcus aureus* arthritis by vaccination with clumping factor A, a novel virulence determinant. J. Infect. Dis. 184, 1572–1580. 10.1086/324430 11740733

[B32] Jung A. L. Stoiber C. Herkt C. E. Schulz C. Bertrams W. Schmeck B. (2016). *Legionella pneumophila*-derived outer membrane vesicles promote bacterial replication in macrophages. PLoS Pathog. 12:e1005592. 10.1371/journal.ppat.1005592 27105429 PMC4841580

[B33] Kafetzis D. A. Maltezou H. C. Constantopoulou I. Antonaki G. Liapi G. Mathioudakis I. (2001). Lack of association between Kawasaki syndrome and infection with *Rickettsia conorii, Rickettsia typhi, Coxiella burnetii* or *Ehrlichia phagocytophila* group. Pediatr. Infect. Dis. J. 20, 703–706. 10.1097/00006454-200107000-00012 11465844

[B34] Kempf V. A. J. Lebiedziejewski M. Alitalo K. Wälzlein J.-H. Ehehalt U. Fiebig J.. (2005). Activation of hypoxia-inducible factor-1 in bacillary angiomatosis. Circulation 111, 1054–1062. 10.1161/01.CIR.0000155608.07691.B7 15723970

[B35] Kempf V. A. J. Volkmann B. Schaller M. Sander C. A. Alitalo K. Rie,ß T.. (2001). Evidence of a leading role for VEGF in *Bartonella henselae*-induced endothelial cell proliferations. Cell. Microbiol. 3, 623–632. 10.1046/j.1462-5822.2001.00144.x 11553014

[B36] Kijek T. M. Mou S. Bachert B. A. Kuehl K. A. Williams J. A. Daye S. P.. (2019). The D-alanyl-d-alanine carboxypeptidase enzyme is essential for virulence in the Schu S4 strain of *Francisella tularensis* and a *dacD* mutant is able to provide protection against a pneumonic challenge. Microb. Pathog. 137:103742. 10.1016/j.micpath.2019.103742 31513897

[B37] Kim H. K. DeDent A. Cheng A. G. McAdow M. Bagnoli F. Missiakas D. M.. (2010). IsdA and IsdB antibodies protect mice against *Staphylococcus aureus* abscess formation and lethal challenge. Vaccine 28, 6382–6392. 10.1016/j.vaccine.2010.02.097 20226248 PMC3095377

[B38] Kingry L. C. Petersen J. M. (2014). Comparative review of *Francisella tularensis* and *Francisella novicida*. Front. Cell. Infect. Microbiol. 4:35 10.3389/fcimb.2014.00035 24660164 PMC3952080

[B39] Kumari B. Saini V. Kaur J. Kaur J. (2020). Rv2037c, a stress induced conserved hypothetical protein of *Mycobacterium tuberculosis*, is a phospholipase: role in cell wall modulation and intracellular survival. Int. J. Biol. Macromol. 153, 817–835. 10.1016/j.ijbiomac.2020.03.037 32165202

[B40] Kundu S. Chakraborty S. (2023). Rhinofacial conidiobolomycosis in an immunocompetent 30-year-old male: a case report. Asian Pac. J. Trop. Med. 16, 329–331. 10.4103/1995-7645.380725

[B41] Larson M. A. Sayood K. Bartling A. M. Meyer J. R. Starr C. Baldwin J.. (2020). Differentiation of *Francisella tularensis* subspecies and subtypes. J. Clin. Microbiol. 58:4. 10.1128/JCM.01495-19 31941692 PMC7098747

[B42] Lee M. Hesek D. Dik D. A. Fishovitz J. Lastochkin E. Boggess B.. (2017). From genome to proteome to elucidation of reactions for all eleven known lytic transglycosylases from *Pseudomonas aeruginosa*. Angew. Chem. Int. Ed Engl. 56, 2735–2739. 10.1002/anie.201611279 28128504 PMC5404236

[B43] Linke D. Riess T. Autenrieth I. B. Lupas A. Kempf V. A. J. (2006). Trimeric autotransporter adhesins: variable structure, common function. Trends Microbiol. 14, 264–270. 10.1016/j.tim.2006.04.005 16678419

[B44] Liu J.-Y. Lin T.-L. Chiu C.-Y. Hsieh P.-F. Lin Y.-T. Lai L.-Y.. (2022). Decolonization of carbapenem-resistant *Klebsiella pneumoniae* from the intestinal microbiota of model mice by phages targeting two surface structures. Front. Microbiol. 13:877074. 10.3389/fmicb.2022.877074 36071974 PMC9441799

[B45] Liu Y. Islam E. A. Jarvis G. A. Gray-Owen S. D. Russell M. W. (2012). *Neisseria gonorrhoeae* selectively suppresses the development of Th1 and Th2 cells, and enhances Th17 cell responses, through TGF-β-dependent mechanisms. Mucosal Immunol. 5, 320–331. 10.1038/mi.2012.12 22354319 PMC3328619

[B46] Low K. L. Rao P. S. S. Shui G. Bendt A. K. Pethe K. Dick T.. (2009). Triacylglycerol utilization is required for regrowth of *in vitro* hypoxic nonreplicating *Mycobacterium bovis* bacillus Calmette-Guerin. J. Bacteriol. 191, 5037–5043. 10.1128/JB.00530-09 19525349 PMC2725574

[B47] Mahajan U. V. Gravgaard J. Turnbull M. Jacobs D. B. McNealy T. L. (2011). Larval exposure to *Francisella tularensis* LVS affects fitness of the mosquito *Culex quinquefasciatus*. FEMS Microbiol. Ecol. 78, 520–530. 10.1111/j.1574-6941.2011.01182.x 22066999

[B48] Malgorzata-Miller G. Heinbockel L. Brandenburg K. van der Meer J. W. M. Netea M. G. Joosten L. A. B. (2016). *Bartonella quintana* lipopolysaccharide (LPS): structure and characteristics of a potent TLR4 antagonist for *in-vitro* and *in-vivo* applications. Sci. Rep. 6:34221. 10.1038/srep34221 27670746 PMC5037446

[B49] Margolis J. J. El-Etr S. Joubert L.-M. Moore E. Robison R. Rasley A.. (2010). Contributions of *Francisella tularensis* subsp. novicida chitinases and Sec secretion system to biofilm formation on chitin. Appl Environ Microbiol., 76, 596–608. 10.1128/AEM.02037-09 19948864 PMC2805214

[B50] Mohammad M. Hu Z. Ali A. Kopparapu P. K. Na M. Jarneborn A.. (2020). The role of *Staphylococcus aureus* lipoproteins in hematogenous septic arthritis. *Sci. Rep*. 10 :7936. 10.1038/s41598-020-64879-4 32404866 PMC7221087

[B51] Mohammad M. Na M. Hu Z. Nguyen M.-T. Kopparapu P. K. Jarneborn A.. (2021). *Staphylococcus aureus* lipoproteins promote abscess formation in mice, shielding bacteria from immune killing. Commun Biol. 4:432. 10.1038/s42003-021-01947-z 33785850 PMC8010101

[B52] Mohammad M. Nguyen M.-T. Engdahl C. Na M. Jarneborn A. Hu Z.. (2019). The YIN and YANG of lipoproteins in developing and preventing infectious arthritis by *Staphylococcus aureus*. PLoS Pathog. 15:e1007877. 10.1371/journal.ppat.1007877 31226163 PMC6608979

[B53] Mosleh I. M. Huber L. A. Steinlein P. Pasquali C. Günther D. Meyer T. F. (1998). *Neisseria gonorrhoeae* porin modulates phagosome maturation. J. Biol. Chem., 273, 35332–35338. 10.1074/jbc.273.52.35332 9857075

[B54] Moss S. M. Taylor I. R. Ruggero D. Gestwicki J. E. Shokat K. M. Mukherjee S. (2019). A *Legionella pneumophila* kinase phosphorylates the Hsp70 chaperone family to inhibit eukaryotic protein synthesis. Cell Host Microbe 25, 454–462.e6. 10.1016/j.chom.2019.01.006 30827827 PMC6529236

[B55] Mulcahy M. E. Geoghegan J. A. Monk I. R. O'Keeffe K. M. Walsh E. J. Foster T. J.. (2012). Nasal colonisation by *Staphylococcus aureus* depends upon clumping factor B binding to the squamous epithelial cell envelope protein loricrin. *PLoS Pathog*. 8 :e1003092. 10.1371/journal.ppat.1003092 23300445 PMC3531522

[B56] Nakamura T. Shimizu T. Inagaki F. Okazaki S. Saha S. S. Uda A.. (2021). Identification of membrane-bound lytic murein transglycosylase A (MltA) as a Growth Factor for *Francisella novicida* in a silkworm infection model. Front. Cell. Infect. Microbiol. 10:581864. 10.3389/fcimb.2020.581864 33553001 PMC7862118

[B57] Navarro L. Koller A. Nordfelth R. Wolf-Watz H. Taylor S. Dixon J. E. (2007). Identification of a molecular target for the *Yersinia* protein kinase A. Mol. Cell. 26, 465–477. 10.1016/j.molcel.2007.04.025 17531806

[B58] Nazarova E. V. Montague C. R. La T. Wilburn K. M. Sukumar N. Lee W.. (2017). Rv3723/LucA coordinates fatty acid and cholesterol uptake in *Mycobacterium tuberculosis*. Elife 6:e26969. 10.7554/eLife.26969.019 28708968 PMC5487216

[B59] Nguyen M. T. Götz F. (2016). Lipoproteins of gram-positive bacteria: key players in the immune response and virulence. Microbiol. Mol. Biol. Rev. 80, 891–903. 10.1128/MMBR.00028-16 27512100 PMC4981669

[B60] Palmqvist N. Foster T. Tarkowski A. Josefsson E. (2002). Protein A is a virulence factor in *Staphylococcus aureus* arthritis and septic death. Microb. Pathog. 33, 239–249. 10.1006/mpat.2002.0533 12473438

[B61] Park B. C. Reese M. Tagliabracci V. S. (2019). Thinking outside of the cell: secreted protein kinases in bacteria, parasites, and mammals. IUBMB Life 71, 749–759. 10.1002/iub.2040 30941842 PMC7967855

[B62] Peltola V. T. McCullers J. A. (2004). Respiratory viruses predisposing to bacterial infections: role of neuraminidase. Pediatr. Infect. Dis. J. 23, S87–S97. 10.1097/01.inf.0000108197.81270.35 14730275

[B63] Quillin S. J. Seifert H. S. (2018). *Neisseria gonorrhoeae* host adaptation and pathogenesis. Nat. Rev. Microbiol. 16, 226–240. 10.1038/nrmicro.2017.169 29430011 PMC6329377

[B64] Raghavan A. Balaka B. Venkatapathy N. Rammohan R. (2020). *Conidiobolus*, a hitherto unidentified pathogen in microbial keratitis. Indian J. Ophthalmol. 68:1461. 10.4103/ijo.IJO_1436_19 32587198 PMC7574109

[B65] Ram S. Cullinane M. Blom A. M. Gulati S. McQuillen D. P. Monks B. G.. (2001). Binding of C4b-binding protein to porin, a molecular mechanism of serum resistance of *Neisseria gonorrhoeae*. J. Exp. Med. 193, 281–296. 10.1084/jem.193.3.281 11157049 PMC2195916

[B66] Ram S. McQuillen D. P. Gulati S. Elkins C. Pangburn M. K. Rice P. A. (1998). Binding of complement factor H to loop 5 of porin protein 1A: a molecular mechanism of serum resistance of nonsialylated *Neisseria gonorrhoeae*. J. Exp. Med. 188, 671–680. 10.1084/jem.188.4.671 9705949 PMC2213355

[B67] Saito S. Quadery F. A. (2018). *Staphylococcus aureus* lipoprotein induces skin inflammation, accompanied with IFN-γ-producing t cell accumulation through dermal dendritic cells. Pathogens 7:64. 10.3390/pathogens7030064 30060633 PMC6161079

[B68] Sarantis H. Gray-Owen S. D. (2012). Defining the roles of human carcinoembryonic antigen-related cellular adhesion molecules during neutrophil responses to *Neisseria gonorrhoeae*. Infect. Immun. 80, 345–358. 10.1128/IAI.05702-11 22064717 PMC3255650

[B69] Sauvage E. Kerff F. Terrak M. Ayala J. A. Charlier P. (2008). The penicillin-binding proteins: structure and role in peptidoglycan biosynthesis. FEMS Microbiol. Rev. 32, 234–258. 10.1111/j.1574-6976.2008.00105.x 18266856

[B70] Schmaler M. Jann N. J. Ferracin F. Landolt L. Z. Biswas L. Götz F.. (2009). Lipoproteins in *Staphylococcus aureus* mediate inflammation by TLR2 and iron-dependent growth *in vivo*. J. Immunol. 182, 7110–7118. 10.4049/jimmunol.0804292 19454708

[B71] Shohet I. Yellin A. Meyerovitch J. Rubinstein E. (1987). Pharmacokinetics and therapeutic efficacy of gentamicin in an experimental pleural empyema rabbit model. Antimicrob. Agents Chemother. 31, 982–985. 10.1128/AAC.31.7.982 3116920 PMC174856

[B72] Siebert C. Villers C. Pavlou G. Touquet B. Yakandawala N. Tardieux I.. (2020). Francisella novicida and F. *philomiragia* biofilm features conditionning fitness in spring water and in presence of antibiotics. PLoS ONE 15:e0228591. 10.1371/journal.pone.0228591 32023304 PMC7001994

[B73] Spatafora J. W. Chang Y. Benny G. L. Lazarus K. Smith M. E. Berbee M. L.. (2016). A phylum-level phylogenetic classification of zygomycete fungi based on genome-scale data. Mycologia 108, 1028–1046. 10.3852/16-042 27738200 PMC6078412

[B74] Spidlova P. Stojkova P. Dankova V. Senitkova I. Santic M. Pinkas D.. (2018). *Francisella tularensis* D-Ala D-Ala carboxypeptidase DacD is involved in intracellular replication and it is necessary for bacterial cell wall integrity. Front. Cell. Infect. Microbiol. 8:111. 10.3389/fcimb.2018.00111 29692981 PMC5903032

[B75] Tegtmeyer N. Neddermann M. Asche C. I. Backert S. (2017). Subversion of host kinases: a key network in cellular signaling hijacked by *Helicobacter pylori* CagA. Mol. Microbiol. 105, 358–372. 10.1111/mmi.13707 28508421

[B76] Valtuille Z. Lefevre-Utile A. Ouldali N. Beyler C. Boizeau P. Dumaine C.. (2023). Calculating the fraction of Kawasaki disease potentially attributable to seasonal pathogens: a time series analysis. EClinicalMed. 61:102078. 10.1016/j.eclinm.2023.102078 37483549 PMC10359724

[B77] van der Weide H. Cossío U. Gracia R. te Welscher Y. M. ten Kate M. T. van der Meijden A.. (2020). Therapeutic efficacy of novel antimicrobial peptide AA139-nanomedicines in a multidrug-resistant *Klebsiella pneumoniae* pneumonia-septicemia model in rats. Antimicrob. Agents Chemother. 64:9. 10.1128/AAC.00517-20 32540976 PMC7449162

[B78] van Heijenoort J. (2011). Peptidoglycan hydrolases of *Escherichia coli*. Microbiol. Mol. Biol. Rev., 75, 636–663. 10.1128/MMBR.00022-11 22126997 PMC3232740

[B79] Vimr E. Lichtensteiger C. (2002). To sialylate, or not to sialylate: that is the question. Trends Microbiol. 10, 254–257. 10.1016/S0966-842X(02)02361-2 12088651

[B80] Voigt K. Cigelnik E. O'donnell K. (1999). Phylogeny and PCR identification of clinically important Zygomycetes based on nuclear ribosomal-DNA sequence data. J. Clin. Microbiol., 37, 3957–3964. 10.1128/JCM.37.12.3957-3964.1999 10565914 PMC85855

[B81] West N. P. Cergol K. M. Xue M. Randall E. J. Britton W. J. Payne R. J. (2011). Inhibitors of an essential mycobacterial cell wall lipase (Rv3802c) as tuberculosis drug leads. Chem. Commun. 47:5166. 10.1039/c0cc05635a 21384024

[B82] Wren J. T. Blevins L. K. Pang B. Basu Roy A. Oliver M. B. Reimche J. L.. (2017). Pneumococcal neuraminidase A (NanA) promotes biofilm formation and synergizes with influenza A virus in nasal colonization and middle ear infection. Infect. Immun., 85(4). 10.1128/IAI.01044-16 28096183 PMC5364304

[B83] Wu S. Ding X. Kong Y. Acharya S. Wu H. Huang C.. (2021). The feature of cervical microbiota associated with the progression of cervical cancer among reproductive females. Gynecol. Oncol., 163, 348–357. 10.1016/j.ygyno.2021.08.016 34503848

[B84] Xiao H. Hu B. Luo R. Hu H. Zhang J. Kuang W.. (2020). Chronic active Epstein–Barr virus infection manifesting as coronary artery aneurysm and uveitis. Virol. J. 17:166. 10.1186/s12985-020-01409-8 33121509 PMC7597064

[B85] Xu Y. Rivas J. M. Brown E. L. Liang X. Höök M. (2004). Virulence potential of the staphylococcal adhesin CNA in experimental arthritis is determined by its affinity for collagen. J. Infect. Dis. 189, 2323–2333. 10.1086/420851 15181582

[B86] Yu Q. Wang L.-C. Di Benigno S. Gray-Owen S. D. Stein D. C. Song W. (2019). *Neisseria gonorrhoeae* infects the heterogeneous epithelia of the human cervix using distinct mechanisms. PLoS Pathog. 15:e1008136. 10.1371/journal.ppat.1008136 31790511 PMC6907876

[B87] Zellner B. Mengin-Lecreulx D. Tully B. Gunning W. T. Booth R. Huntley J. F. (2021). A *Francisella tularensis* L,D-carboxypeptidase plays important roles in cell morphology, envelope integrity, and virulence. Mol. Microbiol. 115, 1357–1378. 10.1111/mmi.14685 33469978

[B88] Zhang X. Zhao Y. Wu Q. Lin J. Fang R. Bi W.. (2019). Zebrafish and galleria mellonella: models to identify the subsequent infection and evaluate the immunological differences in different klebsiella pneumoniae intestinal colonization strains. Front. Microbiol. 10:2750. 10.3389/fmicb.2019.02750 31849893 PMC6900958

[B89] Zhou G. Zhou F. Gu Y. Zhang M. Zhang G. Shen F.. (2022). Vaginal microbial environment skews macrophage polarization and contributes to cervical cancer development. J Immunol Res. 2022, 1–8. 10.1155/2022/3525735 35983073 PMC9381279

